# Circulating nitrate-nitrite reduces oxygen uptake for improving resistance exercise performance after rest time in well-trained CrossFit athletes

**DOI:** 10.1038/s41598-022-13786-x

**Published:** 2022-06-11

**Authors:** Manuel Vicente Garnacho-Castaño, Sergio Sánchez-Nuño, Lorena Molina-Raya, Teresa Carbonell, José Luis Maté-Muñoz, Eulogio Pleguezuelos-Cobo, Noemí Serra-Payá

**Affiliations:** 1 Campus Docent Sant Joan de Déu, Universidad de Barcelona, Carrer de Miret I Sans, 10, 08034 Barcelona, Spain; 2 Department of Cell Biology, Physiology and Immunology, University of Barcelona, Barcelona, Spain; 3 Department of Radiology, Rehabilitation and Physiotherapy, Complutense University of Madrid, Madrid, Spain; 4 Physical Medicine and Rehabilitation Department, Hospital de Mataró, Mataró, Barcelona Spain; 5 School of Health Sciences, TecnoCampus-Pompeu Fabra University, Mataró, Barcelona Spain

**Keywords:** Physiology, Prognostic markers

## Abstract

This study aimed to determine the effects of circulating nitrate plus nitrite (NOx) concentrations on resistance exercise performance, VO_2_ and biomarkers of muscle damage. Eleven well-trained male CrossFit athletes (29.2 ± 3.7 years, 78.9 ± 5.4 kg, 175.1 ± 6.3 cm) carried out a resistance exercise test after drinking 140 mL of beetroot juice (BJ) or placebo. The test consisted of repeating the same resistance exercise routine twice: wall ball shots plus full back squat with 3-min rest (1st routine) or without rest (2nd routine) between the two exercises. Higher NO_x_ plasma levels were verified after BJ than placebo in the pretest and post-test (p < 0.001). A higher number of repetitions was observed after BJ intake compared to placebo in the full back squat exercise during the first routine (p = 0.004). A significantly reduced VO_2_ was detected after BJ intake compared to placebo during rest and full back squat execution in the first routine (p < 0.05). Plasma myoglobin concentrations were significantly increased with BJ compared to placebo (p = 0.036). These results showed that plasma NOx levels reduced VO_2_ after BJ intake during rest time. These reduced VO_2_ was a key factor for improving full back squat performance during the first routine.

## Introduction

Beverages such as beetroot juice (BJ) with a high nitrate (NO_3_^−^) content have become popular among athletes of various exercise modalities due to their biochemical properties for improving physiological function. From a biochemical perspective, dietary inorganic NO_3_^−^ ingested is reduced in the mouth by NO_3_^−^ reductase to nitrite (NO_2_^−^) by commensal anaerobic bacteria. In the stomach, NO_2_^−^ can be reduced to nitric oxide (NO) and other bioactive nitrogen oxides. NO_3_^−^ and the remaining NO_2_^−^ are absorbed from the intestine into the circulation^1^. NO_3_^−^ and NO_2_^−^ can be transformed to bioactive NO in blood and tissues^1^ via reactions with hemoglobin and molybdopterin-containing enzymes^2–4^.

In relation to crucial physiological functions, NO has shown to be a powerful vasodilator^2,5,6^ that increases blood flow especially under acidic and low availability oxygen (O_2_) environments^7^ such as when exercise is performed. Limited O_2_ availability increases the effects of^2,5,6,8^ NO_2_^−^, and the NO derived from NO_2_^−^ is determinant in the compensatory vasodilation, in the delivery of O_2_ to working muscles^9^ and in regulating oxygen uptake (VO_2_)^10^.

Acute and chronic supplementation with dietary NO_3_^−^ has demonstrated to increase plasma concentrations of NO_3_^−^ and NO_2_^−^ and decrease O_2_ cost in endurance-type exercise, at several exercise intensities and using different supplementation protocols^11–14^. In some but not all studies^11,14^, a reduction in pulmonary VO_2_ was not related to an improvement in test performance^15^. Other studies did not find a decrease in VO_2_ and improvements in endurance performance after BJ intake during submaximal exercise^16^, and improvements in performance are even more controversial when referring to well-trained athletes during exercise at light-moderate and high intensity^17^.

Pulmonary VO_2_ closely reflects VO_2_ in exercising muscles^18,19^, and cardiopulmonary exercise testing (CPET) has been considered a fundamental tool to evaluate the effects of NO_3_^−^ intake on the behaviour of VO_2_, especially in endurance-type exercise^14–17^.

The nature of resistance exercise differs from that of a more endurance-type exercise, with local muscle fatigue making it necessary to rest between sets to continue exercising the same muscle mass. In endurance exercise, the effort could be sustained from a few minutes to hours without recovery time. Previous studies by our research group showed that rest was a key factor to maintain exercise tolerance using a vital aerobic energy support when the same resistance exercise was executed^20,21^. We concluded that the half-squat exercise produced a lower VO_2_ than the cycle ergometer exercise at the same relative intensity of the lactate threshold, with both exercises maintaining similar blood lactate concentrations in a predominantly aerobic metabolism. The fact that VO_2_ was lower in resistance exercise compared to endurance exercise and that both types of exercise had similar lactate concentrations could be attributable, at least in part, to the rest time.

Curiously, the transition time from rest to exercise could be a key factor for enhancing high-intensity exercise tolerance due to increased oxidative energy turnover coupled with augmented muscle perfusion in response to NO-induced vasodilation after NO_3_^−^ supplementation^22^. Other nutritional strategies such as L-carnitine L-tartrate supplementation have been shown to reduce muscle tissue oxygenation during occlusion and during recovery from resistance exercise^23^. Carnitine supplementation improved blood flow regulation and oxygen delivery to muscle tissue during and after exercise^24^. Precisely, the vasodilator effect of NO play a key role in the delivery of O_2_ to muscle tissue^9^ and in regulating oxygen uptake (VO_2_)^10^, in an acidic environment with low oxygen availability^7^. It is also tempting to speculate that BJ might be used as a nutritional strategy for decreasing VO_2_ and, consequently, for improving performance during strenuous resistance exercise due to the physiological properties of dietary NO_3_^−^ during rest time.

While most studies have focused on evaluating the effects of dietary NO_3_^−^ on VO_2_ during endurance-type exercise, to the best of our knowledge, no studies have explored the behavior of VO_2_ on exhausting anaerobic-type exercise (resistance exercise) by assessing the effect of recovery time between exercises after acute NO_3_^−^ intake.

The main goal of this study was to determine the effects of acute BJ intake on resistance exercise performance by establishing a causal physiological relationship between NO_3_^−^ plus NO_2_^−^ (NOx) plasma levels and VO_2_ and in well-trained CrossFit athletes. For this purpose, the incidence of rest time between exercises was analyzed. A secondary purpose was to assess the effect of BJ intake on biomarkers of muscle damage. We hypothesized that acute NO_3_^−^ intake could contribute to a reduction in VO_2_ by increasing NO_3_^−^ plus NO_2_^−^ plasma levels and, consequently, improving anaerobic exercise performance after rest time.

## Methods

### Participants

Eleven highly-trained male CrossFit practitioners were recruited for the study (mean ± SD: age = 29.2 ± 3.7 years, body mass = 78.9 ± 5.4 kg, height = 175.1 ± 6.3 cm). The inclusion criteria were: no musculoskeletal injuries or other conditions that could affect test performance; more than two years of experience in CrossFit and resistance exercise training; one-repetition maximum (1RM) in full back squat full back squat greater than 120 kg; regional, national and/or international competition level; no smoking; no consumption of drugs or pharmacological treatment; and no consumption of any other supplement at the time of the study. The participants performed CrossFit workouts at least four times a week.

The sample size was determined from the results of a pilot study involving 10 well-trained students. The calculation of the sample size was carried out as follows: α = 0.05 (5% probability of a type I error) and 1 − β = 0.80 (80% power)^17^. Initially, 12 well-trained CrossFit athletes were recruited for the study. However, one participant was excluded from the final data analysis because he was taking medication/drugs at the time of testing.

Participation was voluntary and according to experimental procedures, each participant provided written informed consent to be included in the study. This investigation was approved by the Ethics Committee of the TecnoCampus, Pompeu Fabra University (Registration number: 56/2019) and was performed according to the principles and policies of the Declaration of Helsinki.

### Procedures

**Figure 1:**
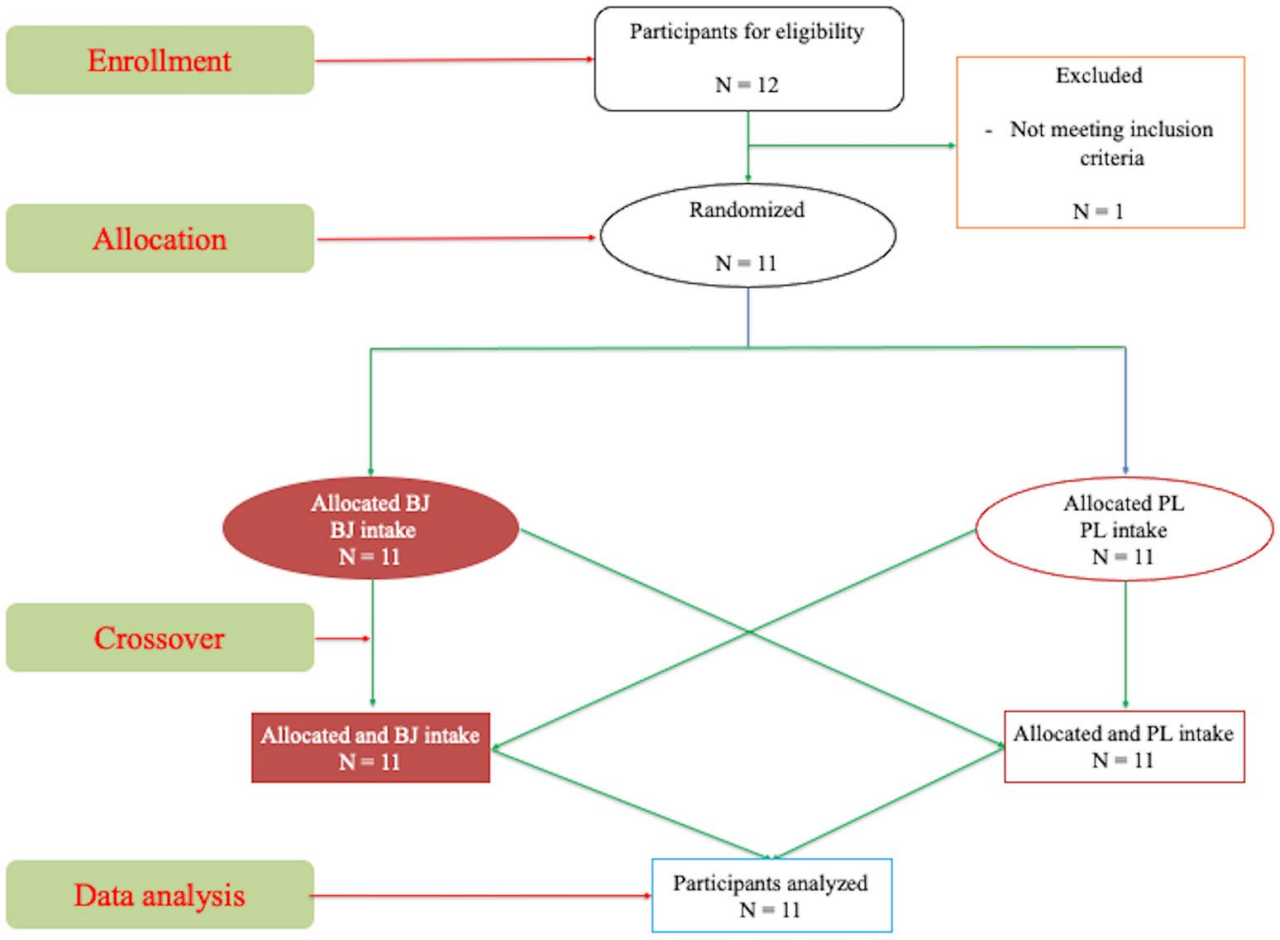
CONSORT flowchart.

The trial was a randomized double-blind, crossover design of three weeks duration. Procedures description and randomization (2 weeks); washout (one-week). Well-trained CrossFit athletes were randomly assigned to BJ or placebo (PL) treatment. Figure 1 shows CONSORT flowchart. Both the participants and the researchers were blinded to the order of treatment (BJ or PL), which was randomized using https://www.randomizer.org.

**Figure 2:**
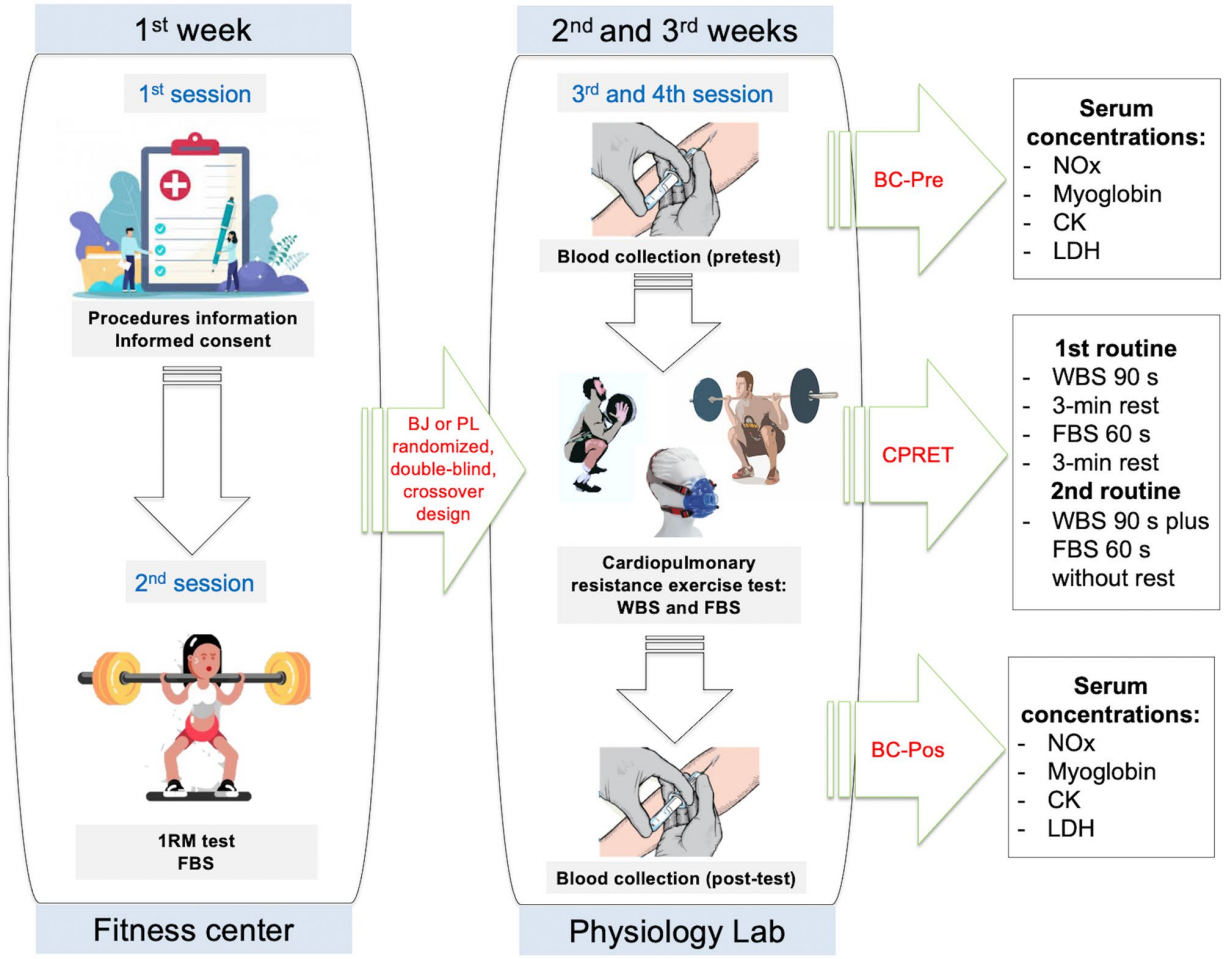
Procedures. 1RM, one-repetition maximum; BC, blood collection; BJ, beetroot juice; CK, creatin kinase; CPRET, cardiopulmonary resistance exercise test; FBS, full back squat; LDH, Lactate dehydrogenase; NOx, nitrate plus nitrite; PL, placebo; WBS, wall ball shots.

Participants attended four testing sessions under similar ambiental conditions (temperature 20–23 °C, relative humidity 45–55%) and at the same time frame (± 30 min) of the day (Fig. 2). Participants were fully familiarized with the experimental procedures.

In the first week (sessions 1 and 2), a researcher explained all the experimental procedures to the athletes (session 1). In the second session, participants carried out a 1RM test in full back squat exercise (see below for details).

Over the next two weeks (sessions 3 and 4), the participants completed the resistance exercise test to compare the two experimental conditions: BJ and PL. A one-week washout period was established between the two sessions^16,17^. Respiratory exchange and heart rate data were recorded during the resistance exercise test.

At rest (pre-test) and after (post-test) the resistance exercise test, blood was collected to determine plasma concentrations of NOx and muscle damage biomarkers (lactate dehydrogenase-LDH, creatine kinase-CK, and myoglobin).

### Resistance exercise tests

From 72 h before the test session until the end of the study, the study subjects did not undertake any high-intensity physical exercise. The participants refrained from any type of physical exercise during the 24 h prior to starting each test session. Participants were allowed to perform regular workouts throughout the study. The workouts allowed were of low intensity (light load resistance training or low intensity aerobic workouts), except during 24 h before starting the test.

In the second session, a 1RM test in full back squat exercise was performed according to the guidelines established by Baechle and Earle^25^. The full back squat test included a general and specific warm-up for all participants. The 1RM test involved a gradual increase in load with an Olympic bar until 1RM was achieved. The 1RM was stated as the maximum weight (kg) lifted by the participant. A 4-min rest was made between each attempt.

In sessions 3 and 4, a resistance exercise test was carried out to compare the two experimental conditions: BJ vs. PL. The resistance exercise test was implemented as in a previous study established by our research group^26^. In summary, two characteristic weightlifting exercises in CrossFit workouts were chosen: Wall ball shots and full back squat. A 10 kg medicine ball was used for the wall ball shots, and the full back squat exercise was performed with free weight (Olympic bar) at a loading intensity of 50% of 1RM. The objective was to reach the highest number of repetitions within a given time domain.

After performing a general and specific warm-up, the resistance exercise test consisted of repeating the same routine twice. The first routine involved 90 s of wall balls plus 60 s of full back squat. A 3-min rest was applied between the two exercises. Afterwards, the second routine involved wall balls for 90 s plus full back squat for 60 s without rest between the two exercises. A 3-min rest was established between the first and the second routine.

Respiratory exchange data were recorded during the resistance exercise test (sessions 3 and 4) using a breath-by-breath open-circuit gas analyzer (Ergostik, Geratherm Respiratory, Bad Kissingen, Germany) which was calibrated before each test according to the manufacturer's instructions. VO_2_, minute ventilation (VE), carbon dioxide production (VCO_2_) and the respiratory exchange ratio (RER) were monitored. The heart rate was checked every 5 s by telemetry (Polar Electro OY, Finland).

### Beetroot juice intake and diet control

BJ or PL was administered 3 h before the start of the resistance exercise test. NO_2_^−^ peak in blood occurs 2–3 h after NO_3_^−^ intake^27^. Both beverages (BJ and PL) were provided in an unlabeled 140 ml garnet-red plastic bottle. The participants were provided with a randomly assigned bottle containing 140 ml (~ 12.8 mmol, ~ 808 mg NO_3_^−^) of BJ Beet-It-Pro Elite Shot concentrate (Beet IT; James White Drinks Ltd. , Ipswich, UK) or PL. The BJ dose was doubled in the present study compared to previous studies by our research group with well-trained triathletes^17^, in order to perform an exhausting resistance exercise protocol with other metabolic characteristics related to a mixed aerobic-anaerobic energy contribution in highly-trained CrossFit practitioners.

The PL beverage was prepared by dissolving 2 g of powdered BJ (~ 0.01 mmol, 0.620 mg of NO_3_^−^, Experience-Naturgreen, Murcia, Spain) in a liter of mineral water. Lemon juice was added to imitate the taste of the commercial supplement. PL and BJ had the same taste. A professional in nutrition and dietetics prepared the PL beverage. ﻿CrossFit athletes were advised of the potential side-effects of BJ, that include the red appearance of urine and feces.

Diet control was established according to the instructions established in previous studies^26,28^. Briefly, nutritional guidelines were established by a dietitian-nutritionist to ensure that CrossFit athletes followed a similar diet 48 h before starting the test which consisted of ~ 60% carbohydrates (5.5 g carbohydrates per kg), 25% lipids, and 15% proteins. The diet was recorded by the athletes in food diaries 48 h before the first resistance exercise test (session 3). The same diet was repeated 48 h before the second test (session 4). Compliance with the dietary guidelines was evaluated by checking the participants' food diaries.

Participants were also provided with a list of foods with high NO_3_^−^ content that they should avoid at least 72 h before the study. ﻿No caffeine, alcohol, or other supplement intake was allowed during the study. Twenty-four hours prior to the test sessions, the participants were asked to refrain from brushing their teeth or using a mouthwash, chewing gum or sweets that could contain a bactericidal substance such as chlorhexidine or xylitol^29^.

### Blood analysis

Three hours after BJ or PL intake (in the morning period), at rest (pre-test) and after (post-test) the resistance exercise test, blood was collected to determine plasma concentrations of NOx and muscle damage biomarkers.

Blood samples were collected from the antecubital vein in a 10 mL EDTA Vacutainer tube. After, serum was obtained, centrifuged at 2500 × g for15 minutes, aliquoted and stored at − 80 °C until posterior analysis.

### Plasma NOx concentrations

NOx levels were measured in plasma previously centrifuged at 14,000 g for 60 min, and ultrafiltered by a 10-kDa cut-off filter (Millipore-Merck, Darmstadt, Germany). Nitrate was converted to nitrite using nitrate reductase, and the total nitrite amount was measured by the Griess reaction using a colorimetric assay kit (Cayman Chemical Co., Ann Arbor, MI, USA). Values were expressed as NOx nmol per mL of plasma.

### Muscle damage biomarkers

Serum concentrations of myoglobin, LDH, and CK were analyzed in an Abbott Architect c8000 analyzer (Abbott Diagnostics, Abbott Laboratories, Abbott Park, IL, USA). Serum myoglobin concentrations were determined by immuno-luminescence methodology. Values were expressed as nmol L^−1^. Serum LDH levels were determined by the lactate to pyruvate method according to International Federation of Clinical Chemistry (IFCC) reference procedures at 37 °C^30^. Values were expressed as U L^−1^. Serum CK concentrations were assessed by the creatine phosphate/nicotinamide adenine dinucleotide phosphate (NADPH)/N-acetylcysteine method according to IFCC reference procedures at 37 °C^31^. Values were expressed as U L^−1^. The coefficient of variation for the between and within assay replicates was less than 5%.

### Statistical analysis

Data were reported as mean and standard deviation (SD), mean and confidence intervals (95% CI) or percentage (%). The Shapiro–Wilk test was applied to check normal distribution of the data. NOx, myoglobin and CK concentrations presented a skewed distribution and were log-transformed to approximate normality. A two-way analysis of variance (ANOVA) with repeated measures was applied (experimental condition x time) to compare the effects of the two experimental conditions (BJ vs. PL). When significant differences emerged, Bonferroni adjustments were implemented for multiple comparisons. The magnitude of the response to both experimental conditions was estimated by partial eta-squared (η_p_^2^). The scale for classification of η_p_^2^ was trivial (< 0.20), small (ηp^2^ = 0.20–0.49), moderate (ηp^2^ = 0.50–0.79), and large (≥ 0.80)^32^.

The formula [(post-test—pretest)/pretest] × 100 was applied to calculate the percentage changes (Δ%) between the pretest and the post-test in muscle damage biomarkers (myoglobin, CK and LDH). The Student’s t-test was applied to identify significant changes in percentage between the two experimental conditions. ﻿The magnitude of the response to BJ or PL conditions was analyzed by the effect size (ES) using Cohen's qualitative descriptors to indicate the changes (small < 0.41, moderate 0.41–0.7, or large > 0.7)^32^.

Significance was set at p < 0.05. All statistical tests were performed using the software package SPSS version 25.0 for Mac (SPSS Inc., Chicago, IL, USA).

### Consent to participate

All participants signed for written informed consent.

## Results

Participants reported drinking BJ or PL at the right time (3 h before the start of the tests). They had to return the bottle. CrossFit athletes maintained their dietary and exercise practices in accordance with the nutritional and exercise guidelines. BJ intake was well tolerated by all participants, with some presenting beeturia (red urine) and red stools.

### Plasma NOx concentrations

**Figure 3:**
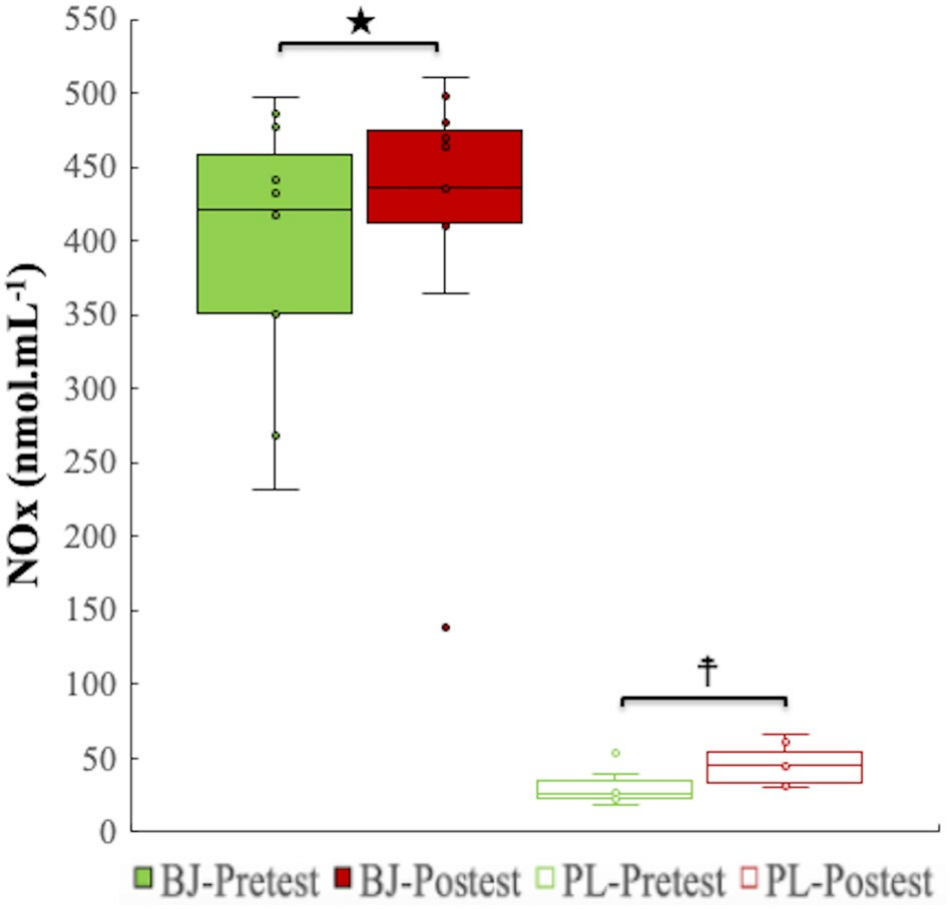
Circulating NOx concentrations in plasma. BJ, beetroot juice; NOx, nitrate plus nitrite; PL, placebo. ★A significant raise in plasma NOx concentrations after BJ intake compared to PL in pretest and posttest (p < 0.001). ☨Significantly higher increase ﻿in post-test than in pretest in PL condition (p < 0.05).

For plasma NOx levels, an interaction, time and experimental condition effects were discovered (p = 0.001, η_p_^2^ = 0.66, SP = 0.98; p = 0.007, η_p_^2^ = 0.53, SP = 0.86; p < 0.001, η_p_^2^ = 0.98, SP = 1.00; respectively). Bonferroni correction showed higher NOx plasma levels after BJ than PL intake in the pretest (BJ: CI 95% 338.70–456.56 mmol mL^−1^; PL: CI 95% 22.61–39.69 mmol mL^−1^) and post-test conditions (BJ: CI 95% 350.94–488.79 mmol mL^−1^; PL: CI 95% 35.37–53.86 mmol mL^−1^) (p < 0.001) (Fig. 3).

### Resistance exercise performance

**Figure 4:**
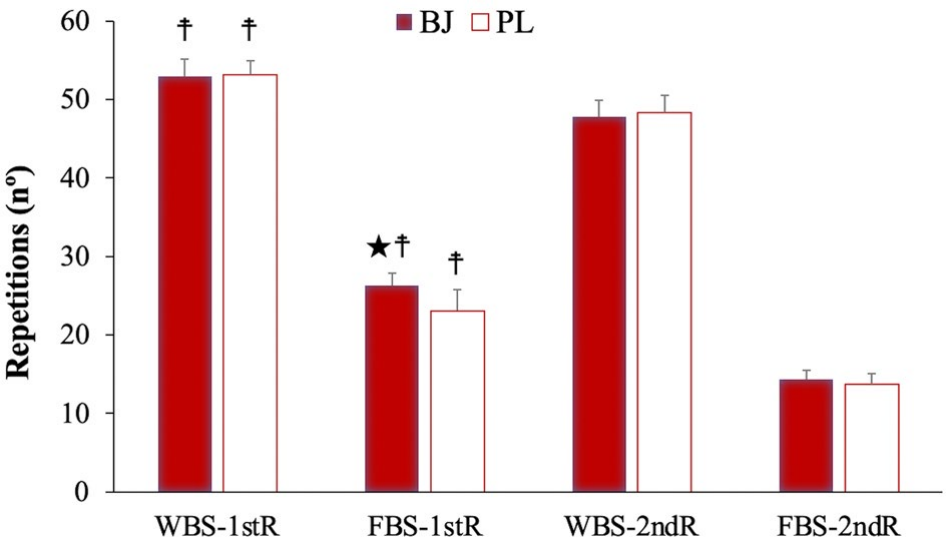
Repetitions completed after beetroot juice and placebo intake. Data are provided as mean and error bars as 95% confidence intervals. 1stR, first routine; 2ndR, second routine; BJ, beetroot juice; FBS, full back squat; PL, placebo; WBS, wall ball shots. ★A significant increase in the FBS repetitions performed after BJ intake compared to PL condition in the first routine (with 3-min rest) (p = 0.004). ☨Repetitions were significantly increased in the WBS and FBS exercises in the first routine compared the second routine (without rest) in the two experimental conditions (p ≤ 0.012).

A significant interaction effect (experimental condition x time) was identified in relation to the number of repetitions performed (p = 0.002, η_p_^2^ = 0.38, SP = 0.93). In addition, significant effects were detected in the time and experimental conditions (p < 0.001, η_p_^2^ = 0.98, SP = 1.00; p = 0.014, η_p_^2^ = 0.47, SP = 0.76; respectively). Bonferroni adjustment determined a higher number of repetitions with BJ compared to PL in the full back squat exercise after rest during the first routine (p = 0.004; BJ: CI 95% 24.65–27.89 repetitions; PL: CI 95% 20.38–25.80 repetitions)). As expected, the number of repetitions was significantly decreased due to muscle fatigue in the second (without rest) (2nd routine–BJ: CI 95% 45.72–49.92 repetitions; PL: CI 95% 46.19–50.54 repetitions) compared to the first routine (with rest) (1st routine–BJ: CI 95% 50.69–55.13 repetitions; PL: CI 95% 51.23–54.95 repetitions) in the wall balls and full back squat (2nd routine–BJ: CI 95% 13.19–15.54 repetitions; PL: CI 95% 12.42–15.04 repetitions) exercises in the two experimental conditions (p ≤ 0.012) (Fig. 4).

### Cardiorespiratory response

**Figure 5:**
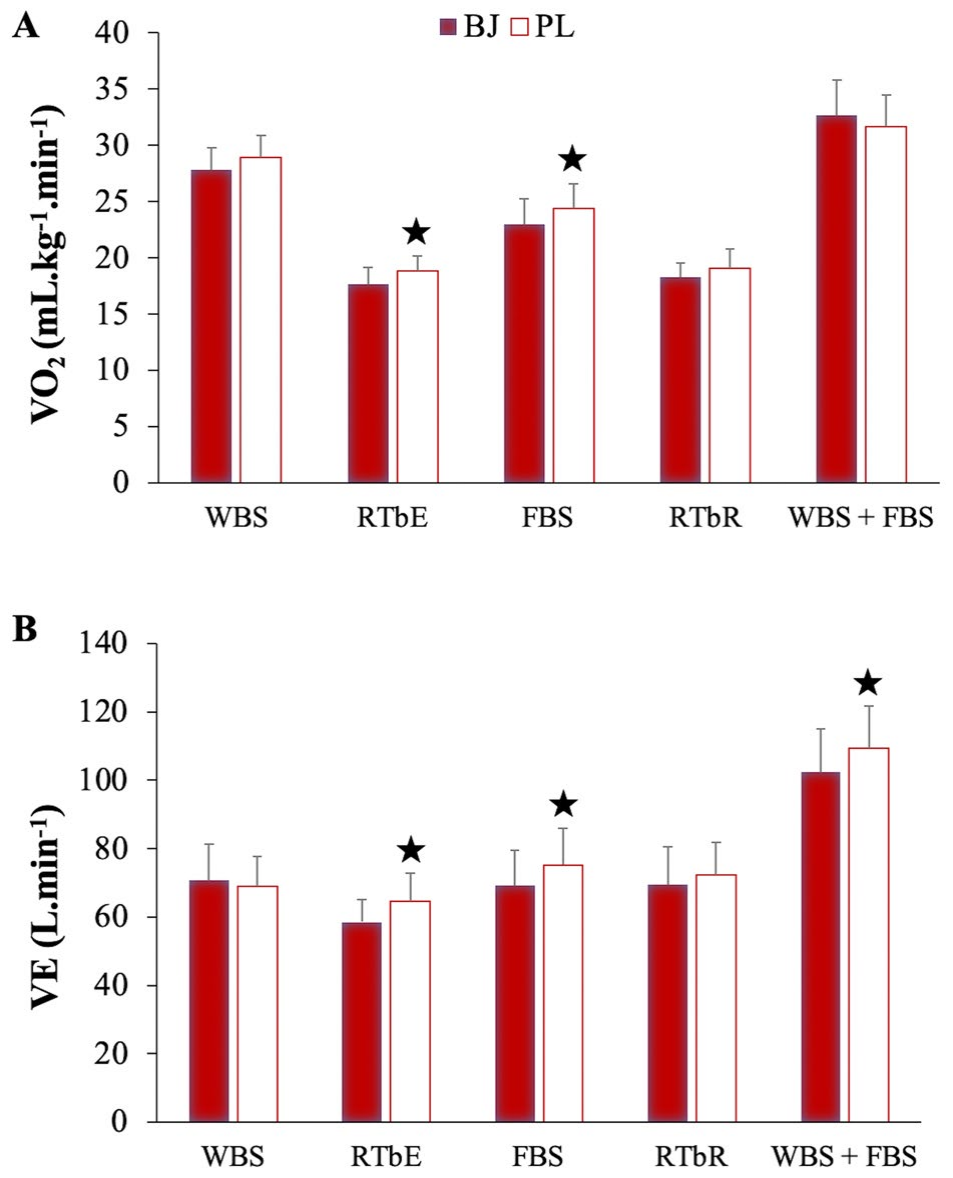
Oxygen uptake (**A**) and ventilation (**B**) after beetroot juice and placebo intake. Data are provided as mean and error bars as 95% confidence intervals. BJ, beetroot juice; FBS, full back squat; PL, placebo; RTbE, rest time between exercises; RTbR, rest time between routines; VE, minute ventilation; VO_2_, oxygen uptake; WBS, wall ball shots. ★A significant increase in the VO_2_ and VE after PL compared to BJ intake (p < 0.05).

For VO_2_, an interaction (experimental condition x time), time and experimental condition effects were detected (p = 0.021, η_p_^2^ = 0.25, SP = 0.78; p < 0.001, η_p_^2^ = 0.94, SP = 1.00; p = 0.007, η_p_^2^ = 0.54, SP = 0.87; respectively). Bonferroni test confirmed a significantly reduced VO_2_ after BJ intake compared to PL during rest (BJ: CI 95% 16.19–19.13 mL kg^−1^ min^−1^; PL: CI 95% 17.61–20.14 mL kg^−1^ min^−1^) and full back squat execution (BJ: CI 95% 20.73–25.28 mL kg^−1^ min^−1^; PL: CI 95% 22.16–26.62 mL kg^−1^ min^−1^) in the first routine (p < 0.05) (Fig. 5A).

In relation to VE, an interaction (experimental condition × time), time and experimental condition effects were noted (p = 0.026, η_p_^2^ = 0.24, SP = 0.76; p < 0.001, η_p_^2^ = 0.84, SP = 1.00; p = 0.017, η_p_^2^ = 0.45, SP = 0.74; respectively). Bonferroni correction verified a significantly decreased VE after BJ intake compared to PL during rest (BJ: CI 95% 52.08–65.01 L min^−1^; PL: CI 95% 56.51–72.8 L min^−1^) and full back squat (BJ: CI 95% 58.97–79.40 L min^−1^; PL: CI 95% 64.62–85.93 L min^−1^) exercise in the first routine (p < 0.05), and during wall balls plus full back squat exercise in the second routine (p = 0.007; BJ: CI 95% 90.13–114.97 L min^−1^; PL: CI 95% 96.91–121.75 L min^−1^) (Fig. 5B).

For RER, a significant interaction effect (experimental condition × time) was verified (p = 0.046, η_p_^2^ = 0.21, SP = 0.69), in addition to a significant time effect (p < 0.001, η_p_^2^ = 0.93, SP = 1.00). No experimental condition effect was observed (p > 0.05). Bonferroni test confirmed a significantly increased RER after BJ intake compared to PL during rest in the second routine (p = 0.010; BJ: CI 95% 1.39–1.51; PL: CI 95% 1.31–1.45).

While no interaction or experimental condition effects were observed for VCO_2_, (p > 0.05), a significant time effect was found (p < 0.001, η_p_^2^ = 0.69, SP = 1.00).

Although no interaction (experimental condition x time) or experimental condition effects were observed for heart rate (p > 0.05), there was a significant time effect (p < 0.001, η_p_^2^ = 0.71, SP = 1.00).

### Muscle damage biomarkers

**Figure 6:**
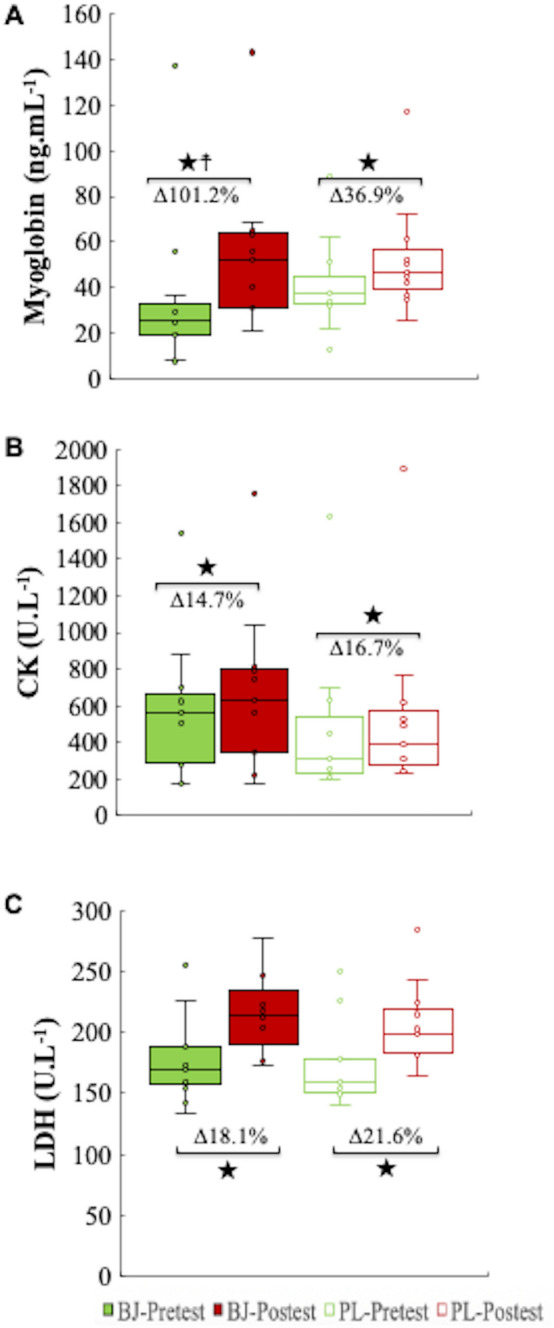
Plasma concentrations of myoglobin (**A**), creatin kinase (**B**) and lactate dehydrogenase (**C**). BJ, beetroot juice; CK, creatin kinase; LDH, lactate dehydrogenase; PL, placebo. ★A significant increase in post-test compared to pretest in the two experimental conditions (p < 0.05). ☨Significantly higher increase after BJ intake (101.2%) than in PL condition 36.9% (p < 0.05).

For serum myoglobin concentrations, an interaction and time effects were identified (p = 0.036, η_p_^2^ = 0.37, SP = 0.59; p = 0.001, η_p_^2^ = 0.72, SP = 0.99; respectively). No experimental condition effect was detected (p > 0.05). Bonferroni adjustment verified a significant increase in myoglobin values in both experimental conditions (p = 0.001, BJ; p = 0.003, PL) (BJ-Pre: CI 95% 11.67–60.15 ng mL^−1^; BJ-Post: CI 95% 32.36–77.28 ng mL^−1^; PL-Pre: CI 95% 27.34–54.84 ng mL^−1^; PL-Post: CI 95% 36.55–69.63 ng mL^−1^). In this regard, myoglobin values were significantly higher with BJ (101.22%) compared to PL (36.9%) (p = 0.036, *d* = 0.93) (Fig. 6A).

No interaction or experimental condition effects were observed for serum CK and LDH concentrations (p > 0.05); however, a significant time effect was detected (p < 0.001, η_p_^2^ = 0.83, SP = 1.00; p < 0.001, η_p_^2^ = 0.77, SP = 0.99; respectively). Significantly higher serum CK (BJ: 14.65%, p = 0.017, *d* = 0.16; PL: 16.70%; p < 0.001, *d* = 0.23) and LDH levels (BJ: 18.11%, p = 0.008, *d* = 0.91; PL: 21.58%, p < 0.001, *d* = 1.10) were observed at the end of exercise under the two experimental conditions. No significant differences (%) were observed in CK and LDH between BJ and PL (p > 0.05) (Fig. 6B,C).

## Discussion

According to our study hypothesis, on comparing BJ with PL, the NOx levels after BJ intake demonstrated a causal physiological relationship between a reduction in VO_2_ during rest time and an improvement in the number of repetitions performed in full back squat exercise (EC main effect). However, no positive effect was observed after BJ ingestion on resistance exercise performance and VO_2_ reduction when the metabolic conditions of exercise (increasingly anaerobic without rest) were changed during the test protocol. It is likely that VO_2_ and resistance exercise performance were affected during rest time by higher NOx concentrations during the two proposed routines. In addition, both experimental conditions raised muscle damage biomarkers. Surprisingly, plasma myoglobin concentrations were significantly increased after BJ compared to PL ingestion.

VO_2_ was reduced in the first routine during the rest time between the wall balls and the full back squat exercises after acute BJ intake. Interestingly, the lower VO_2_ observed in the rest time had a determinant physiological influence on the increase in the number of repetitions in the full back squat exercise exclusively after rest period. The reduction in VO_2_ was maintained during full back squat exercise (only 1st routine) after BJ intake. In contrast, the physiological effects of plasma NOx levels on VO2 and exercise performance diminished in an increased anaerobic environment (2nd routine).

VO_2_ response is not usually evaluated by CPET during strenuous resistance exercises after BJ intake, therefore, this discussion is based on other studies evaluating the same variables in endurance exercise after NO_3_^−^ intake. Cermak et al*.* reported that lower plasma NO_3_^−^ concentrations (30.1 M after 6 days of chronic BJ supplementation) than those found in our study reduced VO_2_ and improved 10-km time-trial performance in trained cyclists^33^. In contrast, Boorsma et al*.* did not find a positive effect on VO_2_ and performance in elite 1500-m runners despite significantly increasing plasma NO_3_^−^ concentrations (from 37 μM to 615 μM) after acute and chronic BJ intake^34^. Other authors described higher NOx concentrations after PL intake (41 μM and 40 μM after 4 and 6 days, respectively) and lower NOx levels after chronic BJ ingestion (147 μM and 159 μM after 4 and 6 days respectively) than those found in our study^35^. Plasma NOx levels were not sufficient stimulus for improving VO_2_ and endurance performance in elite cyclists.

Previously, we demonstrated that recovery time between sets was determinant to sustain resistance exercise tolerance in the same muscle mass by aerobic turnover mechanisms^20,21,36^. The increase in plasma NOx concentrations after BJ intake probably contributed to improving the physiological mechanisms of aerobic energy supply during rest time, enhancing performance (number of repetitions) in the full back squat set. In the first routine proposed, oxidative phosphorylation was the main adenosine triphosphate (ATP)-forming process when wall balls exercise surpasses beyond 60 s (until 90–120 s)^37^, and intramuscular glycogen was the dominant fuel source^38^. O_2_ availability declined during wall balls (1st routine) in both experimental conditions; however, there was a significant trend (p = 0.09) to a reduction in VO_2_ during wall balls exercise after BJ intake. Plasma NOx levels likely produced an advantageous effect, justified at least in part, by a reduced ATP cost for a given rate of muscle contractions during wall balls^11^. A 3-min rest could have been sufficient to stimulate a faster NO_3_^−^ to NO_2_^−^ reduction to NO under an aerobic environment after BJ intake. This suggests that NO-induced vasodilation during rest enhanced oxidative energy turnover coupled with augmented muscle perfusion^22^ which upregulated the efficiency of mitochondrial oxidative phosphorylation^39^. These metabolic and biochemical advantages acquired during rest time were essential for increasing full back squat repetitions at the end of the first routine.

However, when the anaerobic conditions were increased due to muscular demands during the second routine (without rest time between exercises), no positive effect of NOx on VO_2_ and exercise performance was observed. Chronic NO_3_^−^ intake has demonstrated to be a suitable nutritional strategy to enhance resistance exercise performance in terms of total weight lifted, and repetitions until failure at 60% of 1RM^40^. NO_3_^−^ in the potassium NO_3_^−^ salt form did not improve specific performance during CrossFit workouts (Grace protocol) with a mixture of aerobic-anaerobic demand and muscular exhaustion^41^. Ranchal-Sánchez et al. concluded that acute BJ supplementation (120 min prior) improved muscular endurance in back squat exercise but not in bench press without any ergogenic effect on power output and movement velocity in healthy adult men^42^. However, Williams et al. indicated that acute BJ supplementation improved total repetitions, velocity and power during free bench press exercise in resistance-trained male subjects^43^. Perhaps, the differences found between the studies may be due to the resistance exercise protocols used, the dose of BJ (concentration and acute or chronic supplementation), or the level of experience of the participants (healthy or trained). There are limited studies and some controversy over the benefits of BJ intake in resistance exercise performance. VO_2_ assessment is not usual in this type of exercise modalities. The behavior of VO_2_ and the exercise performance observed during both routines indicate a need for more studies to validate the effects of NO_3_^−^ on resistance exercises (with or without rest).

Interestingly, we found that VE was lower after BJ intake compared to PL. Bailey et al*.* showed that chronic BJ supplementation did not alter the VE process during high-intensity knee-extensor exercise in healthy, recreationally active males^11^. In line with their arguments, in our study, VE was not modified by BJ intake during exhausting wall balls exercise (1st routine). Concretely, VE was decreased during the recovery time (1st routine) and the execution of full back squat in the first routine, coinciding with the reduction in VO_2_. Remarkably, there was a trend (p = 0.07) towards a reduction in VCO_2_ during rest in the first routine (aerobic conditions) but not during the execution of the exercises (anaerobic conditions). This suggests that the decrease in VO_2_ induced by NO_3_- during the rest period after exhausting exercise is, at least in part, a consequence of a reduction in the energy cost of respiration and is not exclusive to the muscle contraction process. In fact, this reduction in VE was maintained immediately after the recovery period during full back squat muscle contraction, which allowed improved full back squat performance.

Finally, acute NO_3_^−^ ingestion did not reduce biomarker values for estimating muscle damage assessed by LDH, CK and myoglobin concentrations. As in previous studies on resistance exercises or high intensity eccentric muscle actions^44–46^, the exhausting exercise established here was associated with an increase and release of these enzymes and protein into the blood.

However, plasma myoglobin concentrations were significantly raised after BJ compared to PL ingestion (101% and 37%, respectively). Plasma myoglobin concentration is considered a marker of muscle damage and is usually increased after resistance exercise. Wells et. al demonstrated that plasma myoglobin concentrations were higher after a high-intensity resistance exercise protocol (6 sets, 3–5 repetitions at 90% of 1RM) compared to a high-volume resistance training protocol (6 sets, 10–12 repetitions at 70% of 1RM)^47^. In our study, plasma myoglobin concentrations were increased after BJ intake coinciding with a higher number of repetitions. We found no evidence to clarify whether higher plasma myoglobin concentration is related to higher NOx expression and increased performance, despite the oxygen supply by myoglobin drives oxidative phosphorylation in exercising skeletal muscle^48^. Future studies should focus on the interactions between myoglobin and NO/NO_2_^-^ via dietary NO_3_^-^ consumption and determine how these interactions regulate cellular respiration processes in working muscle.

All these arguments suggest a physiological causal relationship, at least in part, between an increase in plasma NOx concentrations after BJ intake, a lower VO_2_ and VE during the recovery process (first routine) and the coincident increase in full back squat performance immediately after rest (first routine).

This study has some limitations. Although it was a very homogeneous sample, the sample size could be considered small. Therefore, the sample size should be increased in future research as it is usual to observe minimal changes in performance and physiological response in well-trained athletes after BJ intake^17^. The results detected in this study cannot be extrapolated to subjects not trained in CrossFit exercise routines although BJ intake should be considered as nutritional strategy during high intensity CrossFit routines in a predominantly anaerobic metabolism.

## Conclusions

Our results show that plasma NOx levels after BJ intake reduced VO_2_ during rest time and full back squat exercise in the first routine. Plasma myoglobin levels were increased after dietary NO_3_- intake in BJ compared to PL. The physiological causal relationship established between VO_2_, VE and NOx levels likely enhanced full back squat performance in the first routine. This study approach based on rest time opens future lines of research on the role of NO_3_- supplementation in the performance of strenuous effort. Further studies are needed to determine the influence of acute NO_3_^−^ intake on VO_2_ for improving performance in CrossFit workouts or resistance exercise modalities that are prolonged over time.

## Data Availability

All data of this study are available from the corresponding author on reasonable request.
